# High-performance, multi-component epoxy resin simulation for predicting thermo-mechanical property evolution during curing

**DOI:** 10.1038/s41428-025-01022-y

**Published:** 2025-02-10

**Authors:** Sagar Umesh Patil, Josh Kemppainen, Marianna Maiaru, Gregory M. Odegard

**Affiliations:** 1 Michigan Technological University, Houghton, MI USA; 2 Columbia University, New York, NY USA

**Keywords:** Polymers, Mechanical properties

## Abstract

High-performance epoxy systems are extensively used in structural polymer‒matrix composites for aerospace vehicles. The evolution of the thermomechanical properties of these epoxies significantly impacts the evolution of process-induced residual stresses. The corresponding process parameters need to be optimized via multiscale process modeling to minimize the residual stresses and maximize the composite strength and durability. In this study, the thermomechanical properties of a multicomponent epoxy system are predicted via molecular dynamics (MD) simulation as a function of the degree of cure to provide critical property evolution data for process modeling. In addition, the experimentally validated results of this study provide critical insight into MD modeling protocols. Among these insights, harmonic- and Morse-bond-based force fields predict similar mechanical properties. However, simulations with the Morse-bond potential fail at intermediate strain values because of cross-term energy dominance. Additionally, crosslinking simulations should be conducted at the corresponding processing temperature, because the simulation temperature impacts shrinkage evolution significantly. Multiple analysis methods are utilized to process MD heating/cooling data for glass transition temperature prediction, and the results indicate that neither method has a significant advantage. These results are important for effective and comprehensive process modeling within the ICME (Integrated Computational Materials Engineering) and Materials Genome Initiative frameworks.

## Abstract (web summary)

Molecular dynamics simulations were utilized to model and predict thermomechanical properties of a multi-component high-performance epoxy. Virtual curing between the molecules is simulated using REACTER protocol in LAMMPS and LUNAR tool is utilized to assign force field parameters and post-processing of simulated data. Insights into the predicted properties using harmonic- and morse-bond-based force fields, strain-rate sensitivity, glass transition temperature predicted from heating and cooling simulations are provided. The comprehensive set of properties are predicted as a function of processing temperature and crosslinking density required for multi-scale process modeling high-performance epoxy.

## Graphical abstract

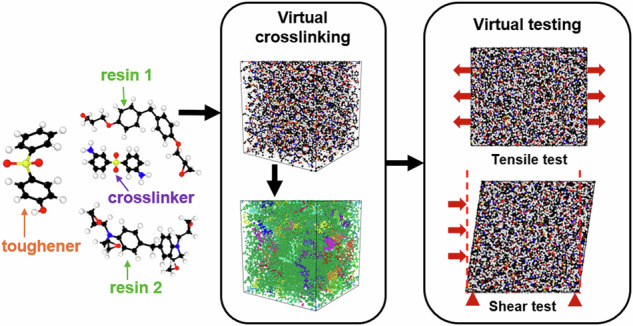

## Introduction

High-performance epoxies are extensively used in polymer matrix composites for aerospace structural components [1]. These epoxies are generally multicomponent systems that consist of a reactive epoxide resin, a crosslinking agent, and multiple additives for tailored material performance [2]. During the fabrication of composite panels, the curing epoxy matrix is subjected to elevated temperatures, resulting in evolving thermomechanical properties, chemical shrinkage, and the formation of residual stresses that further increase upon postprocess cooling. Because these residual stresses are detrimental to the overall performance of composites, quantifying the evolution of properties, shrinkage, and residual stress formation is necessary to optimize composite processing parameters. Experimental characterization of the evolution of composite properties and shrinkage is both time intensive and costly. Consequently, molecular modeling offers a quicker, more cost-effective approach for optimizing the manufacturing of aerospace composites.

Molecular dynamics (MD) simulation is a powerful tool for analyzing molecular structure and behavior and elucidates the effects of temperature, pressure, and other design parameters on the complex molecular structure and properties of epoxies [3, 4]. MD simulations can provide atomistically informed predictions of the evolution of thermomechanical properties and shrinkage during epoxy processing [5–13]. Accurate MD simulations require the correct choice of force fields, which describe the interactions of bonded and nonbonded atoms within the simulation [14–20], and the simulation parameters implemented in the workflow.

Fixed-bond force fields can model covalent bond stretching with either harmonic- or Morse-based energy terms. Harmonic force fields effectively simulate covalent bond stretching with unbreakable springs, whereas Morse stretching energy terms allow for the modeling of bond scission. Li et al. [4, 21] and Odegard et al. [7] demonstrated that the choice of a fixed-bond force field consisting of either a harmonic or Morse-based energy term influences the predicted thermomechanical properties of various epoxy materials. In addition to the force field, careful attention must be given to the selection of simulation parameters such as the strain rate, temperature, and cooling and heating rates to predict the evolution of the thermomechanical properties of epoxies during curing. Patil et al. [10] developed an experimentally validated simulation protocol to predict the properties of a bifunctional epoxy for varying crosslinking densities. The research described above provides a foundation for selecting the force field and simulation parameters to model epoxies composed of only two components: a resin and a hardener. However, a comprehensive understanding of the evolution of material properties in complex epoxies with multiple components is needed to facilitate multiscale process modeling of composite laminates.

This work focuses on the use of MD simulations to accurately predict the physical and thermomechanical properties of a four-component epoxy system at various crosslinking densities. The predicted properties are validated using experimental data from the literature. This approach highlights the accurate modeling and simulation of a multicomponent epoxy, thus addressing the critical need for effectively optimizing next-generation aerospace composites. Other aspects addressed herein include the (a) comparison of the material properties predicted via fixed-bond and reactive force fields, (b) sensitivity of the simulated strain rates to the predicted mechanical properties, (c) comparison of the predicted properties at room and elevated temperatures, (d) application of a viscous-response mapping factor to the mechanical properties, and (e) comparison of the analysis techniques used to predict the thermal properties.

## Material system

**Fig. 1:**
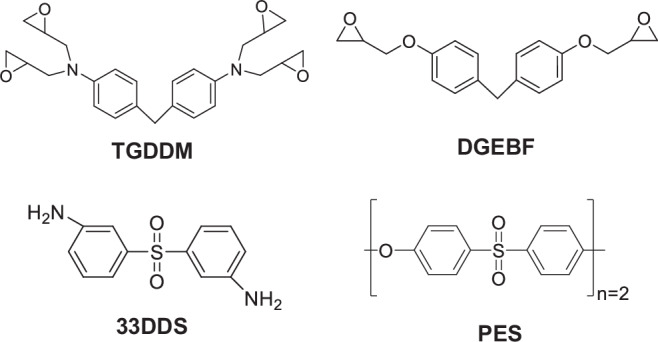
Molecular structures of the monomers. TGDDM and DGEBF are resins, 33DDS is a crosslinker, and PES is a toughening agent

For this study, an epoxy system comprising four chemical components was simulated: N,N,N,N′-tetraglycidyl-4,4′-diaminodiphenylmethane (TGDDM) epoxy resin, diglycidyl ether bisphenol F (DGEBF) epoxy resin, a polyether-sulfone (PES) toughening agent, and a 3,3’- diaminodiphenyl sulfone (33DDS) crosslinker. This epoxy system is similar to the commercial T3900 system, and the components and relative mixtures were obtained from a patent document [22]. The molecular structures of all the constituent monomers are shown in Fig. 1.

## MD simulations

The LAMMPS [23] (Stable Release 2022.04.07 version) (Large-scale Atomic/Molecular Massively Parallel Simulator) software package was used to perform all the MD simulations discussed herein. The OVITO [24] visualization tool was utilized to create snapshots of the MD simulation box. The LUNAR (version 13 May 2024) [25] software package was used to build the MD models and for pre- and postanalysis of the MD simulations. The predicted properties are mass density (*ρ*), total volumetric shrinkage (*ε*_sh_), postgelation volumetric shrinkage, bulk modulus (*K*), shear modulus (*G*), Young’s modulus (*E*), Poisson’s ratio (*ν*), yield strength (*σ*), glass transition temperature (*T*_*g*_), coefficient of thermal expansion (*CTE*), and thermal conductivity (*λ*).

For all the simulations in this work, the Nose‒Hoover barostat [26] and thermostat [27] with a dampening factor of 100 were used to maintain the pressure and temperature of the simulation box with 3D periodic boundary conditions. Lennard‒Jones and Coulomb interactions were given a 12.0 Å cutoff, and weighting factors of 0, 0, and 1 were used for 1–2, 1–3, and 1 − 4 interactions, respectively. Long-range Coulombic interactions are computed using the PPPM solver with the desired relative error in forces within 10^−4^ accuracy.

### Force fields

Two force fields were utilized in this work for property prediction. The interface force field is based on the polymer-consistent force field (PCFF-IFF) [28] and the reactive interface force field (IFF-R) [29]. The PCFF-IFF consists of harmonic bond energy terms, and the IFF-R consists of Morse bond energy terms. The PCFF-IFF was previously used to predict the interfacial mechanical properties of CNT-polymer [14–16] and BNNT-polymer (boron nitride nanotubes) [18, 20], and the IFF-R was shown to predict the accurate properties of thermoset [5, 7, 10] and thermoplastic [19, 30] polymers as a function of processing parameters. First, the PCFF-IFF was used to predict the properties of the epoxy as a function of crosslinking and temperature. Second, the IFF-R was used to predict properties in the fully crosslinked state.

### Model initialization

The number of monomers required to build the MD model was calculated from the part-by-weight values from the patent document [22]. The calculations revealed 189 monomers of TGDDM, 27 monomers of DGEBF, 21 monomers of PES and 99 monomers of 33DDS, resulting in an MD model size of 16,611 atoms. Four individual MD models (replicates) were built to account for statistical deviations in the predicted properties. The MD model of 16,611 atoms is larger than that of 15,000 atoms, which was shown [13] to be sufficient to predict the precise thermomechanical properties of a DGEBF/DETDA epoxy. Once created, the monomers were randomly arranged in a 3D periodic simulation box using the “cell_builder” module in LUNAR. A mixing simulation in the fixed-volume and fixed-temperature (NVT) ensemble for 600 picoseconds (ps) at 327 °C was performed to allow the monomers to mix. The temperature was ramped down to 27 °C within 20 ps after mixing. The procedures outlined above and in the subsequent sections were followed for all four replicates.

### Densification

After mixing, the simulation boxes were densified to a target density of 1.17 g/cm^3^ at 27 °C for 4 ns. During this simulation, the simulation boxes were allowed to compress slowly in the NVT ensemble until the model achieved the target density. After densification, the MD models were subjected to an annealing simulation where the temperature of the simulation box was raised from 27 °C to 327 °C for 20 ps and then cooled from 327 °C to 27 °C at a rate of 75 K/ns in an NVT ensemble. After annealing, a fixed-pressure and fixed-temperature (NPT) ensemble equilibration simulation was performed at 27 °C and 1 atm for 2 ns with 1 fs timesteps to allow the atoms to reconfigure to new equilibrium positions. The mass density and volume of the simulation box were tracked during equilibration.

### Crosslinking

Once the systems were densified and equilibrated, crosslinking simulations were performed between the epoxide carbon in the tetrafunctional TGDDM and bifunctional DGEBF epoxies and the amine nitrogen of the tetrafunctional 33DDS monomer in a two-step reaction [11]. In the first step, the primary amines (-*NH*_*2*_) were converted to secondary amines (-*NH*). In the second reaction, the secondary amines were converted to tertiary amines (-*N-*), with the formation of hydroxyl (-*OH*) groups in both steps. The PES monomers were not modeled to participate in the crosslinking process, because they are nonreactive and used to toughen the cured matrix.

**Fig. 2:**
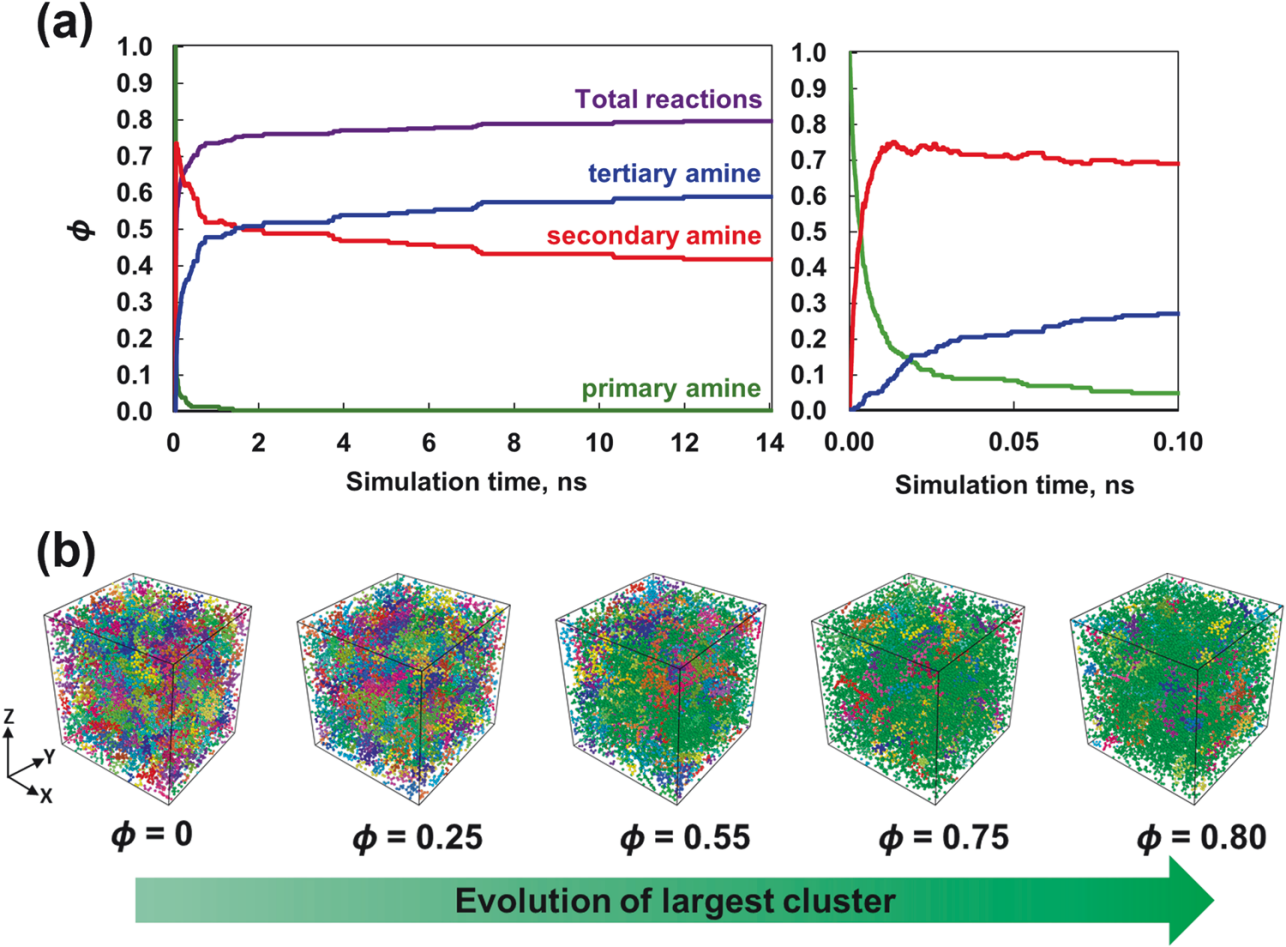
**a** Crosslinking density and number of primary, secondary and tertiary amine reactions vs. simulation time for a representative simulation. **b** Snapshots of a representative simulation box showing different clusters. Each color corresponds to an individual cluster

The REACTER utility developed by Gissinger et al. [31, 32], as implemented in LAMMPS, was used to simulate these reactions in the MD environment. To execute REACTER, each reaction requires three items: a prereaction template that describes the topology of atoms before crosslinking, a postreaction template that describes the topology of atoms after crosslinking, and a mapping file that maps the corresponding atom types in the pre- and postreaction templates. For each replicate, a series of crosslinking densities were established ranging from the uncrosslinked density to the maximum possible achievable value. The crosslinking density (*ϕ*) is the ratio of the total number of covalent bonds formed between the resins and the hardener to the maximum number of covalent bonds that could be formed in the simulated system size. Equal probabilities for all the reactions to occur during the crosslinking simulation were assumed in this study for simplification. As demonstrated by Okabe et al. [33], the activation energy and heat of the reaction associated with the epoxy/amine reaction between TGDDM/33DDS and DGEBF/33DDS can be used as criteria for crosslinking. These are important criterion for simulating crosslinking when the exact chemical reactions are not known in many high-performance polymer systems. The crosslinking simulations were performed at 177 °C (the processing temperature of the epoxy system [34]) and 0.1 fs in the NVT ensemble for 14 ns. To reach 177 °C, the temperature of the equilibrated models from the previous equilibration step was ramped from 27 °C to 177 °C in the NVT ensemble over 20 ps. Figure 2 shows that the run time of 14 ns was sufficient to maximize the total covalent bond formation and reached the highest attainable value of *ϕ* = 0.8. Initially, the total crosslinking density increased at a faster rate and then slowed after reaching *ϕ* = 0.7. Figure 2a also shows the evolution of primary, secondary and tertiary amines as a function of simulation time. Figure 2b shows the growing cluster with increasing crosslinking density, with the largest cluster shown in green. Clusters are defined as groups of connected polymer units in the simulation box. Figure 2b shows that the largest cluster is green and spans the periodic simulation cell at *ϕ* = 0.5, representing an infinite network. Additionally, at *ϕ* = 0.8, the PES molecules and the unreacted epoxy molecules can be uniformly distributed in the simulation cell.

### Equilibration

Following crosslinking, two sets of equilibration simulations were performed. In the first set of equilibration simulations, the temperatures of the crosslinked models were ramped down from 177 °C to 27 °C in the NVT ensemble over 50 ps, and the models were then equilibrated at 27 °C at 1 atm with 1 fs timesteps for 2 ns in the NPT ensemble to prepare the systems for room-temperature property prediction. In the second set of equilibration simulations, the crosslinked models were equilibrated at 177 °C at 1 atm with 1 fs timesteps in the NPT ensemble for 2 ns to prepare the systems for property prediction at the processing temperature. During these equilibration simulations, the mass density and volume of the simulation boxes were tracked, with all the individual energy contributions, to verify the stability of the MD models.

The density and volume of the uncrosslinked and crosslinked models from the equilibration simulations at 27 °C and 177 °C were averaged over the last 500 ps to report *ρ* and *ε*_sh_. *ε*_sh_ was calculated as the change in the volume of the crosslinked model with respect to the uncrosslinked models (*ϕ* = 0).

### Gelation prediction

The gel point marks the transition of the material from a liquid state to a gel-like state at which it is able to sustain a mechanical load. The gel point of the system was predicted by plotting the largest cluster, second-largest cluster, and weight-averaged reduced molecular weight (RMW) of the system as a function of crosslink density. The RMW is the weight-average molecular weight of the reacting groups, with the exception of the largest cluster. Based on these plots, the gel point was determined using three metrics [12, 35, 36]: (1) the inflection point of the molecular weight of the largest cluster (where it dramatically increases), (2) the peak of the molecular weight of the second-largest cluster, and (3) the peak of the RMW of the system. The overall gel point was determined on the basis of the values of these three metrics for each replicate system.

### Free-volume prediction

The free volume is defined as the volume unoccupied by the molecules in the simulation box. During crosslinking, monomers and oligomers move closer together to form covalent bonds, thus increasing the free volume [8, 37]. The temperature influences the molecular motion of the simulation, which also affects the free volume evolution [8]. Therefore, the free volume evolution with crosslinking density and temperature was predicted using the “free_volume” module in LUNAR for each replicate. For the free volume calculations, a voxel size of 0.25 Å, a probe diameter of 1.1 Å, a 3D periodic box, the “numba‒ddp” run mode, which utilizes domain decomposition, and the spherical shell method were used.

### Mechanical simulations

Multiple mechanical deformation simulations were performed in this work. First, to determine the sensitivity of the predicted *E* and *σ* to the applied strain rates, a set of tensile simulations in the three principal directions (*x*, *y*, *z*) of the MD simulation box in the fully crosslinked (*ϕ* = 0.8) state were performed with the PCFF-IFF. These tensile simulations utilized the “*fix deform*” command in LAMMPS with a 10% strain in the NPT ensemble at 27 °C with 0.1 fs timesteps. These simulations were performed for all four replicates and four strain rates of 2 × 10^9^, 2 × 10^8^, 2 × 10^7^, and 2 × 10^6 ^s^−1^. The corresponding stress‒strain plots from these simulations were used to calculate *E* and *σ* from the linear region, as described by Pisani et al. [38].

Second, to predict the mechanical properties with PCFF-IFF at various crosslinking densities (*ϕ* = 0 to 0.8), a series of simulations were performed as outlined by Odegard et al. [7]. *K* was predicted from equilibration simulations at two pressure settings of 1 atm and 5000 atm in the NPT ensemble with 1 fs timesteps at 27 °C. The ratio of the change in pressure and volume was used to predict *K* for each MD model. To predict *G*, shear deformations in the three principal planes (*xy*, *yz*, and *xz*) were simulated using the “*fix deform*” and “*triclinic*” commands in LAMMPS in the NPT ensemble at a 2 × 10^8 ^s^−1^ strain rate, 10% total strain, 27 °C temperature, and 0.1 fs timesteps. The (*K*, *G*) pair was then used to predict the (*E*, *ν*) pair using the standard relations for linear-elastic isotropic materials [39]. The *σ* values were predicted using the von Mises stress calculated from the shear deformations [7]. For completeness, all the corresponding equations are included in the SI (Sec. S1).

The mechanical properties predicted with MD need to be corrected in some circumstances because of the viscoelastic nature of polymers and their corresponding response to high orders-of-magnitude simulated strain rates [6, 7, 9]. In this study, a phenomenological approach developed by Patil et al. [9] to map the MD-predicted mechanical properties to laboratory-scale properties was utilized to correct the *G* and *σ* values, and then, the corresponding *E* and *ν* values were calculated from the (*K*, *G*) pair. The details of the viscous response mapping are included in the SI (Sec. S2).

### Thermal simulations

To predict the *T*_*g*_ and *CTE* of the epoxy system, the MD model in the fully crosslinked state (*ϕ* = 0.8) was subjected to heating and cooling rates of 25 K/ns, 50 K/ns and 75 K/ns. For the heating cycle, the temperature of the simulation box was elevated from −73 °C to 427 °C. For the cooling cycle, the temperature was increased from 427 °C to −73 °C. Both cycles were run in the NPT ensemble with 1 atm pressure and 0.1 fs timesteps. Different heating and cooling rates were used in this work to determine the sensitivity of these parameters to the predicted thermal properties. The changes in simulation box volumes were monitored during these simulations. The corresponding volume−temperature (V − T) plots were used to predict the *T*_*g*_ values using three different analysis methods from the LUNAR package:

1. The hyperbola-based method of Patrone et al. [40], where the V − T plot is fitted with hyperbolas in the low- and high-temperature regions until the tangent to the hyperbolas is 90%, converges to the linear regression curve fit in the high- and low-temperature regions. The intersection point of these tangents is the *T*_*g*_ of the epoxy.
2. The piecewise regression (segmented) method [38] was implemented using the pwlf [41] package in Python (version 3.11) to calculate the optimal breakpoint, which was the *T*_*g*_ of epoxy.
3. The bilinear regression method, where the data in the V − T plot at high-temperature (277 °C to 427 °C) and low-temperature (−173 °C to −23 °C) regions are fitted with a linear regression model in Python (version 3.11). The intersection point of the two linear lines is the *T*_*g*_ of the epoxy.

For all three methods, the slopes of the linear lines in the high- and low-temperature regions and those of the tangents were used to obtain the *CTE* above and below the *T*_*g*_ at constant pressure.

To predict *λ* at 27 °C and 177 °C, nonequilibrium molecular dynamics (NEMD) simulations [42] were implemented using the methods and parameters described by Odegard et al. [8]. A temperature difference of 40 °C was maintained between the heat source and the heat sink, with the mean temperature of 27 °C and 177 °C (for room temperature and high temperature, respectively). The heat flux was then calculated in the fixed-volume and fixed-energy (NVE) ensemble for 2 ns with 1 fs timesteps. The equations used to calculate *λ* are provided in the SI (S3).

### Property prediction using the IFF-R

To predict the properties of the fully crosslinked epoxy (*ϕ* = 0.8) using IFF-R, the equilibrated MD models at *ϕ* = 0 and *ϕ* = 0.8 for all four replicates from PCFF-IFF were converted to IFF-R using the “auto_morse_bond_update” module in LUNAR. After this conversion, the models were equilibrated at 27 °C with 1 atm pressure and 1 fs timesteps for 2 ns in the NPT ensemble. During this equilibration, the density and volume of the simulation box were tracked. The predicted *ρ* and *ε*_sh_ values are reported above. To predict *E*, *ν*, and *σ*, tensile simulations in the fully crosslinked state (*ϕ* = 0.8) in the three principal directions (*x*, *y*, *z*) were performed. These tensile simulations utilized the “*fix deform*” command in LAMMPS with a 2 × 10^9 ^s^−1^ strain rate and 10% axial strain in the NPT ensemble at 27 °C and 0.1 fs timesteps. The *K* values were predicted from equilibration simulations at two pressure settings of 1 atm and 5000 atm with the NPT ensemble at 27 °C and 1 fs timesteps. The *G* values were calculated from the (*E*, *ν*) pair using the standard relationships for linear-elastic isotropic materials [39]. To predict *T*_*g*_, *CTE*, and *λ* with the IFF-R, the steps outlined in the above sections were implemented.

## Results

This section details the predicted thermomechanical properties of the epoxy system as a function of crosslinking density and temperature.

### Comparison of the PCFF-IFF and IFF-R

Table 1 presents the predicted thermomechanical properties of the epoxy system at *ϕ* = 0.8 and 27 °C obtained from the PCFF-IFF and IFF-R force fields. The predicted properties of the modeled epoxy using both force fields agree with each other and with the experimental data from the literature. The *E* values of the epoxy are in good agreement with the experimental values of similar aerospace grade 977-3 epoxies. The predicted *σ* values from both force fields agree, with no experimental values obtained for similar multicomponent epoxy systems in the literature. However, the MD-predicted *σ* at *ϕ* = 0.8 of the modeled epoxy is significantly greater than the experimentally measured ultimate tensile strength of the 977-3 (90 MPa) epoxy. This finding suggests that MD overpredicts the yield strength of the epoxy, because the experimental yield strength is always lower than the tensile strength. These observations suggest that this epoxy does not have a significant strain rate effect on the predicted *E* values but has a significant strain rate effect on the predicted *σ* values, which arises from the order-of-magnitude discrepancy in the MD strain rates (2 × 10^9^ s^−1^) and experimental strain rates (1 × 10^−3^ s^−1^) [6, 7, 10]. All the other mechanical and thermal properties from both the PCFF-IFF and IFF-R agree with each other and with the experimental values. The results indicate that under the given modeling settings (2 × 10^9^ s^−1^ strain rate, 10% axial strain, and 50 K/ns cooling rate), the implementation of Morse bonds in the IFF-R does not significantly influence the predicted properties of the modeled epoxy.

**Table 1:** Predicted properties of fully crosslinked epoxy

Property	Units	PCFF-IFF	IFF-R	Experimental
*ρ*	g/cm^3^	1.239 ± 0.002	1.235 ± 0.003	1.220–1.250 [34]
*ε*_*sh*_	%	4.4 ± 0.2	4.2 ± 0.3	3.5–4.5 [58]
*E*	GPa	4.9 ± 0.6	4.3 ± 0.6	3.7 [52]
*ν*	—	0.33 ± 0.06	0.26 ± 0.07	0.35 [52]
*K*	GPa	6.29 ± 0.17	6.28 ± 0.23	—
*G*	GPa	1.18 ± 0.03	1.10 ± 0.13	1.37 [52]
*σ*	MPa	130.64 ± 15.62	143.49 ± 12.90	—
*T*_*g*_	°C	183.34 ± 16.35	180.27 ± 11.32	204 [34]
*CTE above T*_*g*_	10^−5^/°C	21.97 ± 0.43	18.56 ± 0.34	21.17 [53]
*CTE below T*_*g*_	10^−5^/°C	10.77 ± 0.40	9.06 ± 0.36	9.14 [53]
*λ*	W/m.K	0.22 ± 0.01	0.23 ± 0.01	0.24 [54], 0.20 [55]

**Fig. 3:**
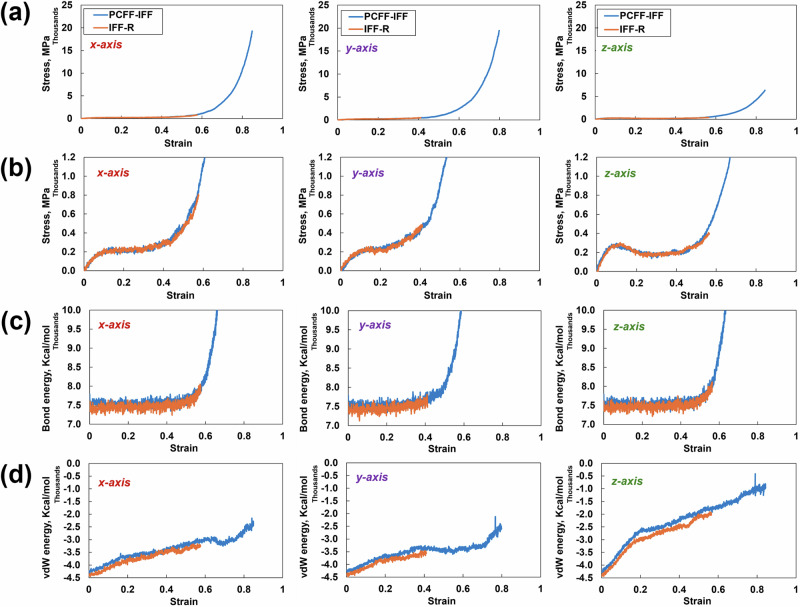
**a** Stress‒strain plot from the tensile simulation in all three directions, **b** zoomed-in view of the stress‒strain plots, **c** corresponding bond energies, and **d** corresponding vdw energies

To understand whether the predicted stress‒strain curves from the two force fields show agreement for very large strains (>15%), the tensile simulations were strained to 90% for one replicate at *ϕ* = 0.8 using both force fields in all three cartesian coordinate directions. The results of these simulations are presented in Fig. 3. No significant difference is observed in the stress‒strain response, bond energy values, or vdW energy values between the two force fields in all three directions. However, while the tensile simulation in the PCFF-IFF ran successfully, the IFF-R simulations failed at strains between 40% and 60%. The simulation failures were due to excessive velocities originating from large instantaneous energy terms, compromising atom tracking between simulation subdomains. Both Gallegos et al. [43] and Delasoudas et al. [44] made the same observation about the IFF-R at large strain values for a crosslinked phenolic system (40% strain) and the bifunctional DGEBF/DETDA epoxy (180% strain), respectively. These authors attributed the unstable simulations with IFF-R at large strains to the high atomic velocities generated by the unconstrained Class II harmonic cross terms. In other words, the Morse bond allows the standard bonding potential to disassociate. However, the harmonic cross terms induce large energies during significantly large deformations, thus resulting in excessive atomic velocities.

### Strain rate sensitivity

**Fig. 4:**
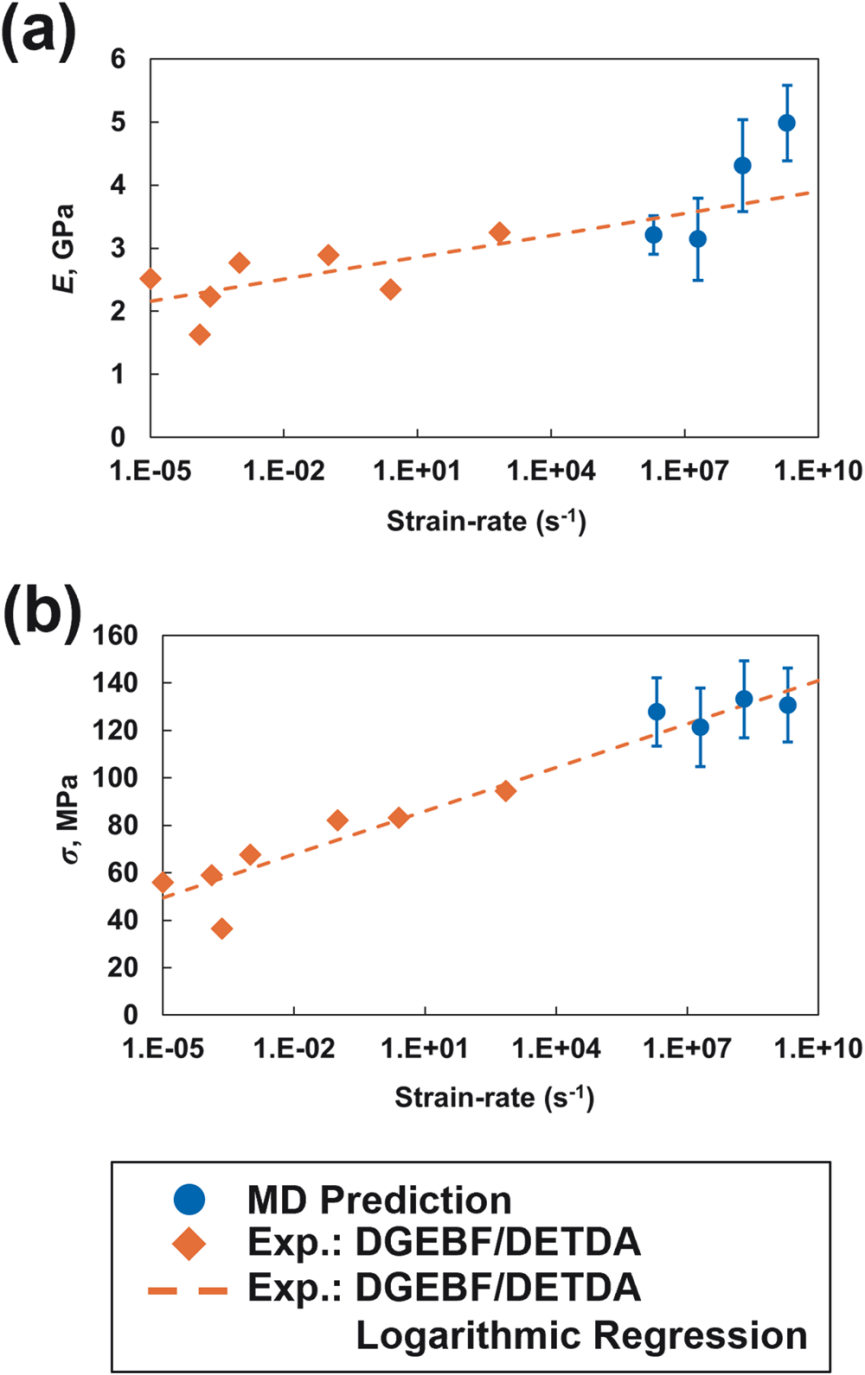
**a** Young’s modulus and **b** yield strength vs. strain rate on a logarithmic scale

Figure 4 shows the MD-predicted *E* and *σ* values of the epoxy system as a function of the strain rate compared with the experimental values of DGEBF/DETDA epoxy [45, 46]. As expected, the experimental E and *σ* values increase with increasing strain rates. A comparison with a bifunctional DGEBF/DETDA epoxy was performed in this study because of the lack of experimental data in the literature for multicomponent epoxies at a range of strain rates. The many order-of-magnitude differences between the experimental and MD strain rates are due to the high computational cost associated with MD simulations [6, 7, 11]. Nevertheless, the MD-predicted *E* and *σ* values from all four strain rates increase with increasing strain rate, thus following a similar trend as the experimental measurements and agreeing with the extrapolated logarithm regression of the experimental data.

### Physical properties

**Fig. 5:**
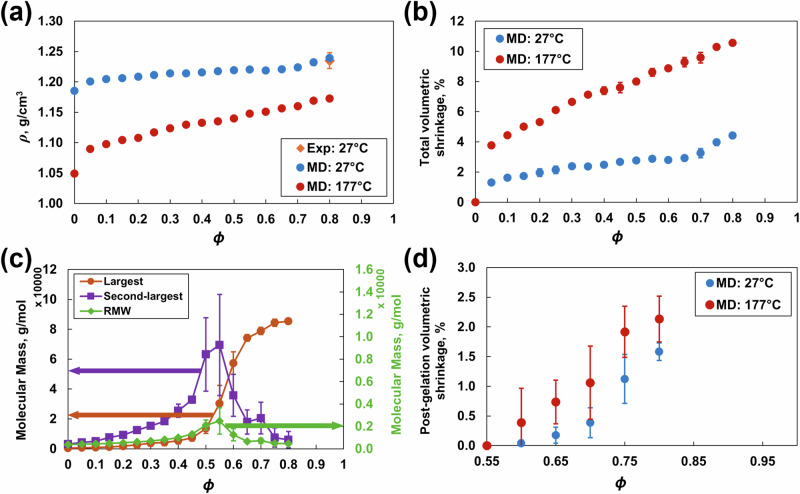
**a** Mass density, **b** total volumetric shrinkage, **c** molecular mass, and **d** postgelation volumetric shrinkage vs. crosslinking density and temperature. The error bars represent the standard deviation associated with the four replicate predictions

Figure 5a shows the mass density as a function of *ϕ* at 27 °C and 177 °C. The predicted mass density at 27 °C and *ϕ* = 0.8 is 1.239 ± 0.002 g/cm^3^. This value agrees with the experimental value of 1.22–1.25 g/cm^3^ for the T3900 epoxy system [34]. The mass density gradually increases with increasing crosslink density because of increased network connectivity due to the formation of covalent bonds between the molecules at both temperatures. The mass density at 177 °C is lower than that at 27 °C because of thermal expansion.

Figure 5b shows the total volumetric shrinkage as a function of crosslinking density at 27 °C and 177 °C. The predicted shrinkage at 27 °C and *ϕ* = 0.8 is 4.23 ± 0.25%. This finding agrees with the shrinkage observed during the experimental curing of epoxy resins [4]. At both temperatures, the volumetric shrinkage increases with increasing crosslink density because of the formation of covalent bonds that pull the molecular network into a tighter configuration.

Figure 5c shows the gel points calculated using the three metrics: the molecular mass of the largest cluster, the second-largest cluster and the RMW for all four replicates. The gel point for this epoxy system was 0.55, which agrees with the experimental value of 0.50–0.55 reported in the literature [47].

Figure 5d shows the postgelation volumetric shrinkage, which is calculated as the change in volume of the crosslinked model with respect to the volume at the gel point (*ϕ* = 0.55). The predicted postgelation volumetric shrinkage at *ϕ* = 0.8 is 1.58 ± 0.13% at 27 °C and 2.13 ± 0.41% at 177 °C. This value agrees with the experimentally measured value of 2.36 ± 0.08% for DGEBF/DETDA epoxy [10]. The postgelation volumetric shrinkage increases nonlinearly with increasing crosslinking density at 27 °C and linearly with increasing crosslinking density at 177 °C. These trends agree with other experimental observations for other epoxy systems [48, 49]. The prediction of the evolution of postgelation volumetric shrinkage is vital, because it dominates the development of residual stresses during curing [50, 51].

### Free volume

**Fig. 6:**
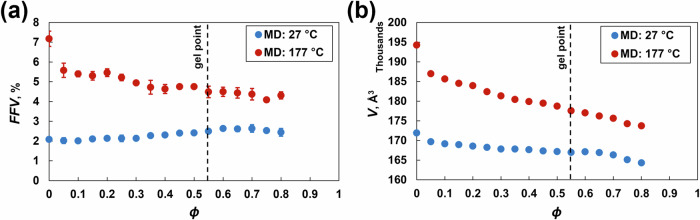
**a** Fractional free volume and **b** simulation box volume at 27 °C and 177 °C vs. crosslinking density

Figure 6a, b show the fractional free volume (FFV) and simulation box volume as a function of *ϕ* at 27 °C and 177 °C. The FFV at 27 °C did not significantly change with increasing crosslinking density. At 177 °C, the FFV decreases dramatically until *ϕ* = 0.30 and then decreases steadily with increasing crosslinking density. Both the FFV and simulation box volume are greater at elevated temperatures because the volumetric expansion is driven by the coefficient of thermal expansion (Fig. 6a, b). The change in the volume of the simulation box because of the motion of the molecules at varying temperatures corresponds directly to the changes in FFV (Fig. 6b).

### Mechanical properties

**Fig. 7:**
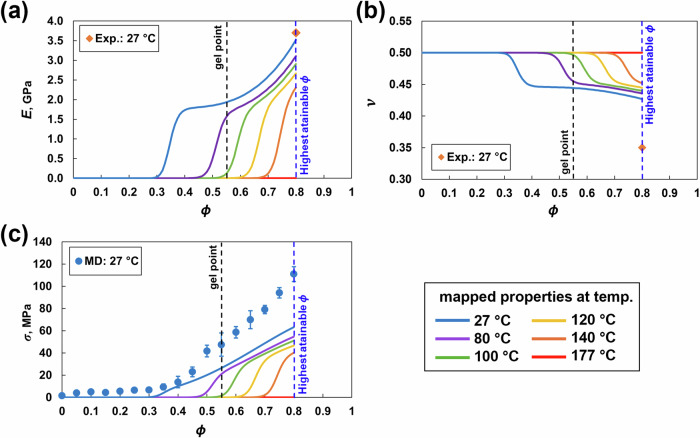
MD mechanical properties mapped via the viscous response equation [9]: **a** Young’s modulus, **b** Poisson’s ratio and **c** yield strength vs. ϕ and temperature

Figure 7 shows the mapped mechanical properties as a function of *ϕ* and temperature. The predicted *K* and *G* viscous mapping is included in the SI (S2). The predicted *E* value in Fig. 7a at *ϕ* = 0.8 was 3.58 ± 0.26 GPa, which agrees with the corresponding experimental value of 3.70 GPa for 977-3 epoxy [52], suggesting that there is no significant strain rate effect observed in the predictions for the epoxy system analyzed in this paper. The stiffness increases with increasing crosslinking because the growing branched network can sustain a greater load. With increasing temperature, the stiffness decreased and was zero at the processing temperature. The decrease in stiffness is likely because of the increased mobility of atoms, which results in increased interatomic distance and hence reduced interatomic forces. The predicted *ν* value of 0.40 ± 0.01 at *ϕ* = 0.8 in Fig. 7b is slightly greater than the experimental value of 977-3 epoxy [52]. With increasing crosslinking, *ν* decreases because the growing crosslinked network restricts lateral deformation when axial loads are applied. With increasing temperature, *ν* increases because of increased molecular mobility and reaches 0.5, similar to an incompressible liquid. Figure 7c shows the predicted *σ* of 111.01 ± 6.67 MPa at *ϕ* = 0.8. The *σ* values increase with increasing crosslinking and are relatively low in the pregelation regime (*ϕ* = 0 to 0.55), because the material is a viscous liquid and cannot sustain significant mechanical loads below the gel point (*ϕ* = 0.55). Moreover, the σ values increase sharply in the postgelation regime.

### Thermal properties

**Fig. 8:**
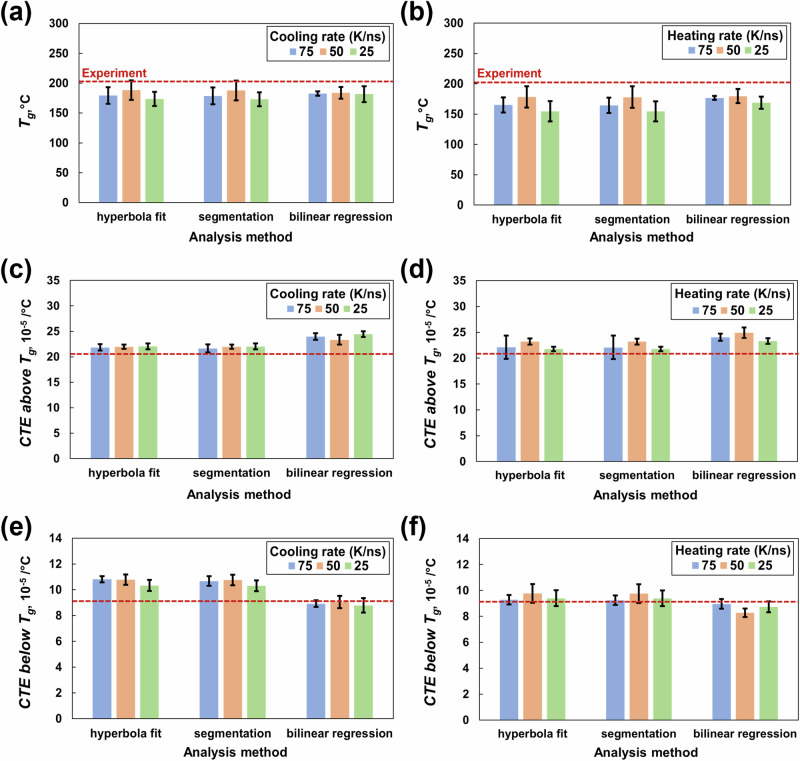
Glass transition temperature from **a** cooling and **b** heating simulations, CTE above the T_g_ from **c** cooling and **d** heating simulations, and CTE below the T_g_ from **e** cooling and **f** heating simulations. The experimental data are shown by the dotted red line

Figure 8a, b present the *T*_*g*_ for the fully crosslinked state (*ϕ* = 0.8). In general, all three analysis methods compute comparable (within standard deviations) *T*_*g*_ values. The *T*_*g*_ values predicted from the cooling (Fig. 8a) and heating (Fig. 8b) rates of 50 K/ns agree with the experimental values of T3900 epoxy [34].

Figure 8c, f show the CTE values both above and below the *T*_*g*_ for the fully crosslinked state (*ϕ* = 0.8). For the CTE above the *T*_*g*_, the hyperbolic fit and segmentation methods predict comparable (within 5%) values that agree with the experimental values for a toughened epoxy system [53] for all three cooling rates (Fig. 8c) and heating rates (Fig. 8d).

For the CTE below the *T*_*g*_, the linear regression methods predict a value that agrees with the experimental value for a toughened epoxy system for all three cooling rates (Fig. 8e). For heating rates (Fig. 8f) of 75 and 25 K/ns, the CTE values below *T*_*g*_ agree with the experimental values [53] for all three analysis methods. The predicted CTEs both above and below the *T*_*g*_ are compared with the experimental values of a toughened epoxy system [53], which differs from the model in this study. Hence, a difference in values is expected.

**Fig. 9:**
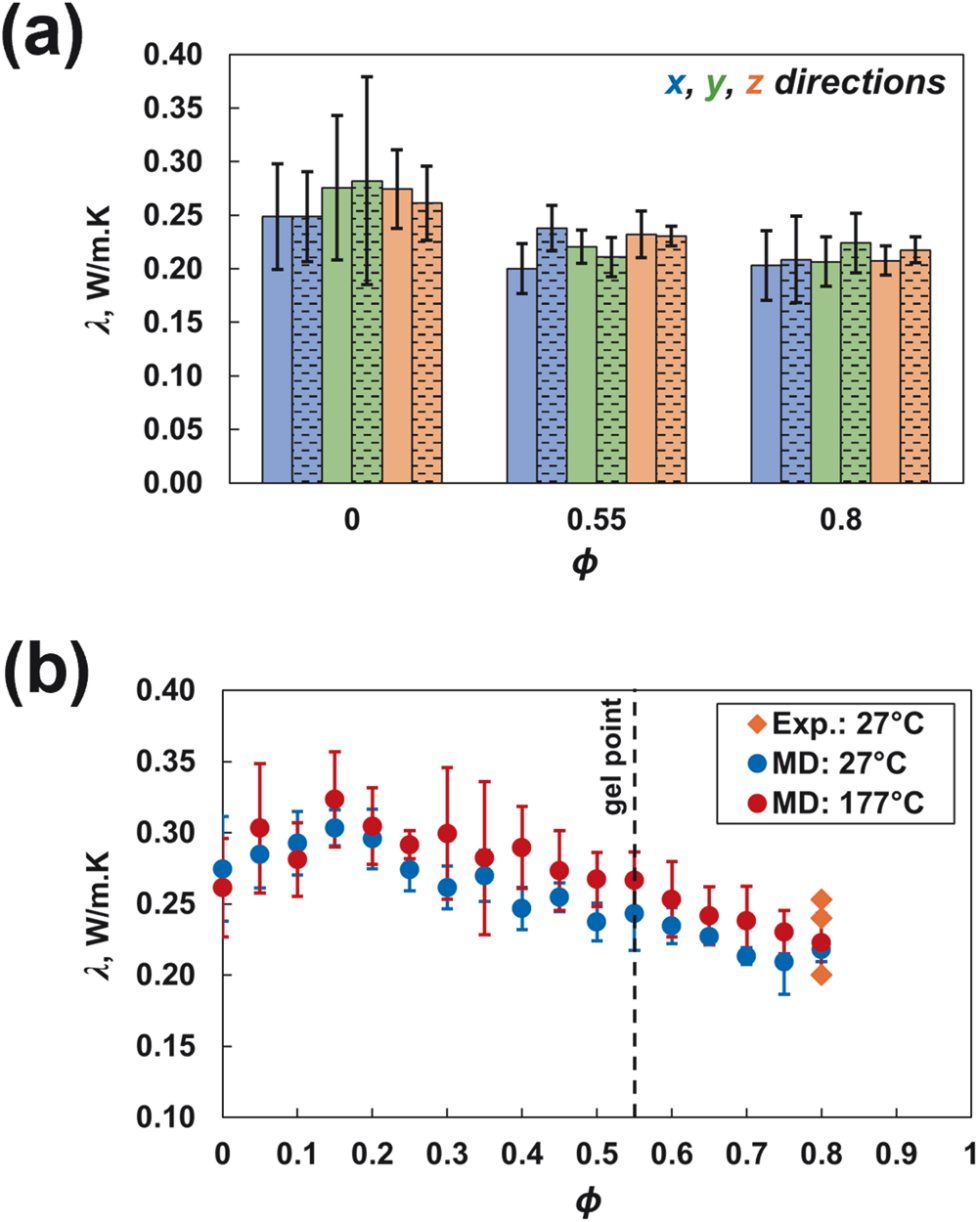
Thermal conductivity in the **a** x-, y- and z-directions for a representative ϕ and temperature (27 °C are represented by solid bars; 177 °C are represented by patterned bars) and in the **b** z-direction vs. ϕ and temperature

Figure 9a shows that the *λ* values predicted in all three principal directions for the uncrosslinked state (*ϕ* = 0), gel point (*ϕ* = 0.55), and fully crosslinked state (*ϕ* = 0.8) do not change significantly (all within standard deviations) for both 27 °C and 177 °C in the *x*, *y* and *z* principal directions. Therefore, *λ* was predicted only in the *z* direction for all other crosslinking densities, as shown in Fig. 9b. The *λ* values at *ϕ* = 0.8 and both temperatures agree with the experimental values for a similar toughened epoxy system [54–56]. The change in temperature has no significant effect on the predicted *λ*. Wan et al. [42] reported the same trend for DGEBF/DETDA epoxy. For both 27 °C and 177 °C, *λ* slightly increases initially until *ϕ* = 0.15 and then decreases gradually with increasing crosslinking. Additionally, higher standard deviations were observed for predictions at 177 °C because of thermal fluctuations and increased molecular mobility at higher temperatures. The results presented in this work depend on the composition and simulation settings chosen for modeling. Consistent with previously published work [57] on the influence of stoichiometry on the predicted properties of the DGEBF/DETDA system, the effects of the size of the PES molecule and stoichiometry on the predicted properties of multicomponent epoxies will be discussed in the future.

## Conclusions

The predicted thermomechanical properties of the multicomponent epoxy system considered in this study generally match the available experimental values from the literature, thus validating the modeling method. This study provides the following important observations:

- The results indicate that the predicted elastic properties below the gel point need to be corrected (via a previously established technique [9]) to accurately capture the heavy viscoelastic response that is not accessible with all-atom MD simulations.
- The harmonic bond force field (PCFF-IFF) and Morse bond force field (IFF-R) predict similar elastic properties and yield strengths. However, MD simulations conducted with the IFF-R tend to fail at intermediate strain values because of the high-energy dominance of the class II cross terms at relatively large bond stretches.
- The predicted postgelation shrinkage is greater for processing temperature simulations than for room temperature simulations, emphasizing the importance of simulating the curing process at the corresponding processing temperature for accurate shrinkage prediction.
- The use of cooling or heating simulations has little influence on the prediction of *T*_*g*_ and thermal expansion, considering the statistical standard deviation observed with MD simulations. The experimentally observed effects of the cooling rate on the *T*_*g*_ measurement are smaller than this standard deviation. Thus, the use of cooling rate correction factors does not substantially improve the accuracy of *T*_*g*_ predictions with MD. This conclusion is consistent with previous observations [8].
- The method of analyzing heating/cooling data from MD simulations (hyperbola fit, segmentation, and bilinear regression) does not significantly influence the predicted *T*_*g*_ values.
- The predicted thermal conductivity of this system is not significantly affected by the temperature within the 27 to 177 °C range.

These results are important for the prediction of multicomponent epoxy resin properties at the molecular level and are crucial inputs for effective and comprehensive process modeling within ICME and Materials Genome Initiative frameworks.

## Supplementary information

Supplementary Information

## Data Availability

The data that support the findings of this study are available from the corresponding author upon reasonable request. The LUNAR (version 13May2024) package is available for public download from a GitHub repository with the link “https://github.com/CMMRLab/LUNAR”.
