# Supplementary material for: High-performance, multi-component epoxy resin simulation for predicting thermo-mechanical property evolution during curing
Source: Polym J. 2025 Feb 10;57(5):539–52. doi: 10.1038/s41428-025-01022-y (PMC12055604; doi:10.1038/s41428-025-01022-y)
Supplement: Supplementary file 1 — Supplementary Information [file 41428_2025_1022_MOESM1_ESM.docx]

Supplementary Material

**High-Performance, Multi-Component Epoxy Resin Simulation for Predicting Thermo-Mechanical Property Evolution During Curing**

Sagar Umesh Patil^1*^, Josh Kemppainen^1^, Marianna Maiaru^2^, Gregory M. Odegard^1^

*^1^Michigan Technological University, Houghton, MI-49931, USA*

*^2^Columbia University, New York, NY-10025, USA*

*^*^Corresponding author: spatil4@mtu.edu*

# Mechanical Property Prediction Equations:

The bulk modulus (*K*) was predicted by subjecting all the MD models at each crosslink density to an NPT simulation at 300 K and 5000 atm for 1 ns and another NPT simulation at 300 K and 1 atm for 1 ns. The volume (*V*) was recorded during these simulations and the *K* values were calculated as described elsewhere [1] using Equation S1. The shear modulus (*G*) was predicted from shear deformations in the *yz*, *xy*, *xz* planes [2] at 300 K and a strain rate of 2×10^8^ s^-1^. The corresponding shear stress-strain curve was plotted for each replicate and shearing plane and a bilinear breakpoint [3] was determined by observing the strain at which the slope changed significantly. The shear modulus was calculated as the slope of the linear line before the breakpoint. The Young’s moduli (*E*) and Poisson’s ratios (𝜈) for each MD model were determined from the corresponding values of bulk modulus and the average shear modulus using standard isotopic elasticity equations [4] as shown in Equation S2 and S3.

$$\begin{aligned} K={-V}_{O}\left( \frac{dP}{dV} \right)\#\left( S1 \right) \end{aligned}$$

$$\begin{aligned} E=\frac{9KG}{3K+G}\#\left( S2 \right) \end{aligned}$$

$$\begin{aligned} \nu=\frac{3K-2G}{2(3K+G)}\#\left( S3 \right) \end{aligned}$$

The yield strength was predicted from the von Mises stress (*σ_vM_*) from the individual stress components obtained from the shear deformation simulations using Equation S4:

|  |  | $\begin{aligned} \sigma_{vM}=\sqrt{\frac{1}{2}\left[ \left( \sigma_{x}-\sigma_{y} \right)^{2}+\left( \sigma_{y}-\sigma_{z} \right)^{2}+\left( \sigma_{y}-\sigma_{x} \right)^{2}+6\left( {\tau_{xy}}^{2}+{\tau_{xz}}^{2}+{\tau_{yz}}^{2} \right) \right]}\#\left( S4 \right) \end{aligned}$ | | |
| --- | --- | --- | --- | --- |
|  | | |  |  |

The von Mises stress-shear strain curve was plotted, and the yield strength was the corresponding stress at the same breakpoints as described above for the shear modulus. The yield strength was determined for each replicate and shearing plane.

# Viscous Response Mapping:

It has been established that for the modeled epoxy system there is a significant strain-rate effect in predicted *E* and *σ* [3, 5]. The same applies to *G*. Patil et al. [6] developed a simple method to correct the predicted elastic modulus of thermosets to account for the viscoelastic effects in terms of strain rate (*α*), degree of cure (𝛽), and temperature (*τ*),

$$\begin{aligned} \frac{E}{E_{MD}}=f_{\alpha}\left( \alpha\right)f_{\beta}\left( \beta\right)f_{\tau}\left( \tau,\beta\right)\#\left( S5 \right) \end{aligned}$$

where *E* is the laboratory-scale Young’s modulus and *E_MD_* is the MD-predicted Young’s modulus. Each of the scalar functions *f*_𝛼_, *f*_ϕ_, and *f*_𝜏_ are functions of non-dimensional variables and are valued between 0 and 1. This method involves seven phenomenological parameters, and the corresponding parameters established for a bifunctional DGEBF/DETDA are given in Table 2.

Table 2: phenomenological parameters [6] implemented to correct predicted *E* and *σ*

| **Material parameters** | *α_a_* | *α_b_* | $\beta$*_0_* | $\beta$*_σ_* | *τ_₀_^a^* | *τ_₀_^b^* | *τ_⁎_^a^* | *τ_⁎_^b^* | *τ_σ_* |
| --- | --- | --- | --- | --- | --- | --- | --- | --- | --- |
| **DGEBF/DETDA** | 0.0147 | 1.0849 | 0.45 | 0.020 | -0.4712 | 0.5268 | 1.40 | 0.300 | 0.009 |

Although the original use of this mapping was only applied to modulus [6], it was assumed herein that the same mapping applied to the shear modulus and yield strength. Furthermore, the strain rate correction term, *f_𝛂_*(*𝛂*), was set to 1 because the strain rate response is highly dependent on the specific epoxy formulation [3, 5] and the high-performance system considered herein is likely to exhibit a strain rate response that is significantly smaller than that of the epoxy system studied by Patil et al [6].

Fig. S1 shows the mapped *K* and *G* as a function of *ϕ* and temperature. The experimental values of K at 27°C was calculated from the experimental values of *E* and *G* from toughened epoxies like 977-3 and 3501-6 epoxy [7]. No viscous response mapping was done for *K*, because the viscous response of thermoset materials is only apparent in deformations with a finite deviatoric (shape changing) component of deformation. For hydrostatic (volume changing) deformations, the response is purely elastic [8]. The prediction of *K* with MD is important as it is difficult to measure it experimentally. The mapped (*E*, 𝜈 ) was predicted using (*K*, *G*) pair in Fig. 8a and 8b in main manuscript. As the temperature increases, the corrected *G* is zero below gel point as the material is viscous. The properties decrease as the temperature increases. At 177°C (the processing temperature for this epoxy system), the modulus is expected to reduce to zero because the material is in a rubbery state and unable to sustain any substantial mechanical load.


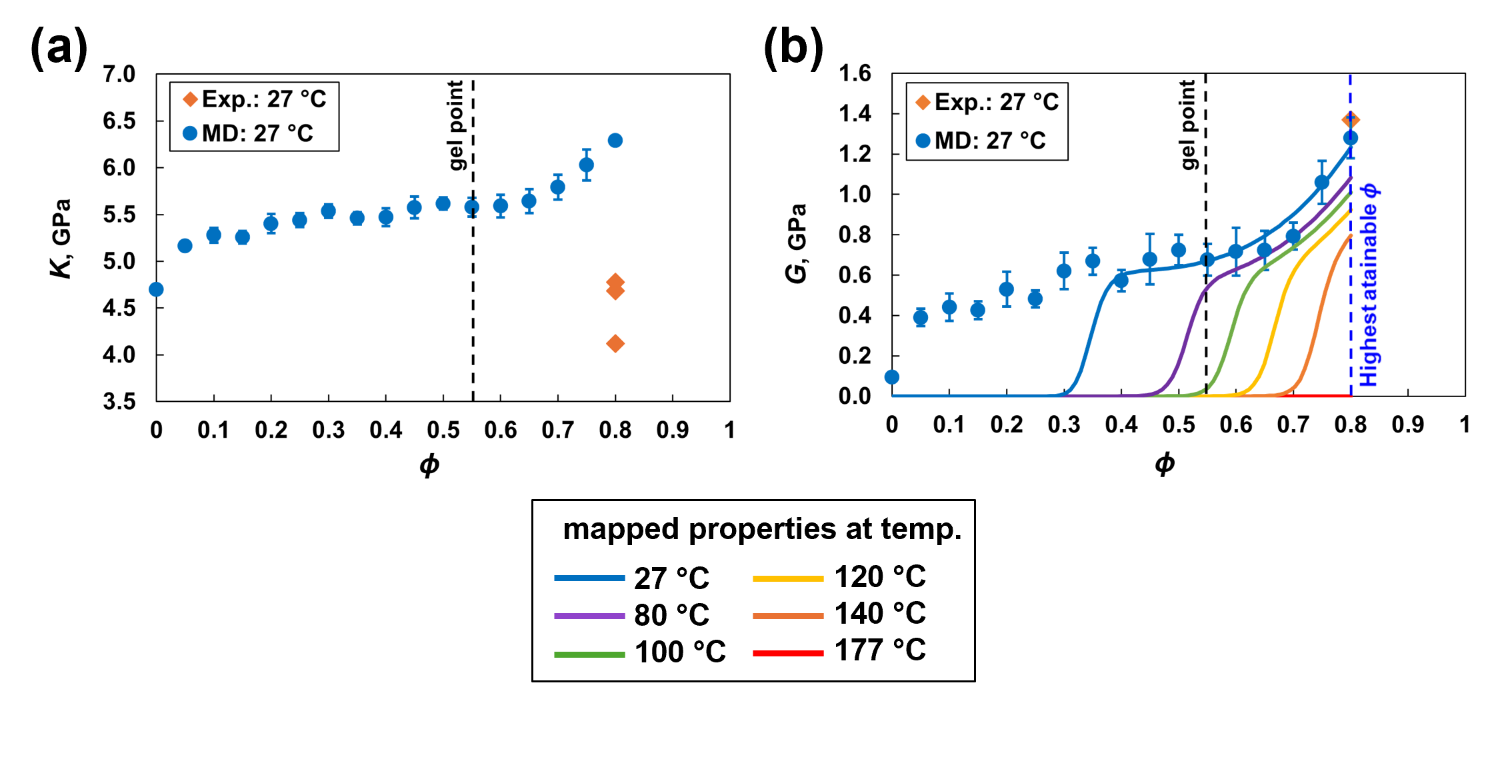


**Fig. S1** (a) Bulk modulus, and (*b*) mapped shear modulus vs *ϕ* and temperature. The solid circles represent uncorrected MD predictions.

# Thermal Conductivity Prediction Equations:

The thermal conductivity is calculated from the simulation outputs from LAMMPS,

$$\begin{aligned} \lambda=-\frac{J}{\nabla T}\#\left( S6 \right) \end{aligned}$$

$$\begin{aligned} J=\frac{1}{2S}\left( \left| \frac{dQ_{in}}{dt} \right|+\left| \frac{dQ_{out}}{dt} \right| \right), \#\left( S7 \right) \end{aligned}$$

Where, $J$ is he heat flux, $\nabla T$ is the temperature gradient, $S$ is the cross-sectional area of the simulation box, $dQ_{in}$ and $dQ_{out}$ are the energies extracted from the heat sink or added to the heat source respectively. The temperature gradient is obtained by linear fitting to the local temperature, excluding the temperature points at heat bath and fixed boundary. More details are provided elsewhere [9].

**References**

1. Tack, J.L.,Ford, D.M., *Thermodynamic and mechanical properties of epoxy resin DGEBF crosslinked with DETDA by molecular dynamics.* Journal of Molecular Graphics & Modelling, 2008. **26**(8): p. 1269-1275.

2. Al Mahmud, H., Radue, M.S., Chinkanjanarot, S., Pisani, W.A., Gowtham, S., Odegard, G.M., *Multiscale Modeling of Carbon Fiber- Graphene Nanoplatelet-Epoxy Hybrid Composites using a Reactive Force Field.* Composites Part B-Engineering, 2019. **172**: p. 628-635.

3. Odegard, G.M., Jensen, B.D., Gowtham, S., Wu, J., He, J., Zhang, Z., *Predicting mechanical response of crosslinked epoxy using ReaxFF.* Chemical Physics Letters, 2014. **591**: p. 175-178.

4. Malvern, L.E., *Introduction to the Mechanics of a Continuous Medium*. 1969, Upper Saddle River, NJ: Prentice-Hall, Inc.

5. Odegard, G.M., Patil, S.U., Deshpande, P.P., Kanhaiya, K., Winetrout, J.J., Heinz, H., Shah, S.P., Maiaru, M., *Molecular Dynamics Modeling of Epoxy Resins Using the Reactive Interface Force Field.* Macromolecules, 2021. **54**(21): p. 9815-9824.

6. Patil, S.U., Krieg, A.S., Odegard, L.K., Yadav, U., King, J.A., Maiaru, M., Odegard, G.M., *Simple and convenient mapping of molecular dynamics mechanical property predictions of bisphenol-F epoxy for strain rate, temperature, and degree of cure.* Soft Matter, 2023. **19**(35): p. 6731-6742.

7. Isaac M. Daniel, O.I., *Engineering Mechanics of Composite Materials*. Second ed. 2006: Oxford University Press.

8. Flory, P.J., *Principles of polymer chemistry*. 1953: Ithaca : Cornell University Press.

9. Wan, X., Demir, B., An, M., Walsh, T.R., Yang, N., *Thermal conductivities and mechanical properties of epoxy resin as a function of the degree of cross-linking.* International Journal of Heat and Mass Transfer, 2021. **180**: p. 121821.
